# FX ENTRAIN: scientific context, study design, and biomarker driven brain-computer interfaces in neurodevelopmental conditions

**DOI:** 10.3389/fnins.2025.1618804

**Published:** 2025-09-09

**Authors:** Jae Citarella, Peyton Siekierski, Lauren Ethridge, Grace Westerkamp, Yanchen Liu, Elizabeth Blank, Lynxie Voorhees, Laura Batterink, Stephanie R. Jones, Elizabeth Smith, Debra L. Reisinger, Meredith Nelson, Devin K. Binder, Khaleel A. Razak, Makoto Miyakoshi, Steve Wu, Donald Gilbert, Paul S. Horn, Lisa A. De Stefano, Craig A. Erickson, Ernest V. Pedapati

**Affiliations:** ^1^Neurobehavioral Treatment Discovery Team, Cincinnati Children’s Research Foundation, Division of Child and Adolescent Psychiatry, Cincinnati Children’s Hospital Medical Center, Cincinnati, OH, United States; ^2^University of Cincinnati, Cincinnati, OH, United States; ^3^Section on Developmental and Behavioral Pediatrics, Department of Pediatrics, University of Oklahoma Health Sciences Center, Oklahoma City, OK, United States; ^4^Department of Psychology, University of Oklahoma, Norman, OK, United States; ^5^Department of Psychology, Western Centre for Brain and Mind, Western Institute for Neuroscience, University of Western Ontario, London, ON, Canada; ^6^Department of Neuroscience, Brown University, Providence, RI, United States; ^7^Carney Institute for Brain Science, Brown University, Providence, RI, United States; ^8^Department of Pediatrics, University of Cincinnati College of Medicine, Cincinnati, OH, United States; ^9^Division of Biomedical Sciences, School of Medicine, University of California, Riverside, CA, United States; ^10^Psychology Department, University of California, Riverside, Riverside, CA, United States; ^11^Department of Psychiatry, University of Cincinnati College of Medicine, Cincinnati, OH, United States; ^12^Division of Neurology, Cincinnati Children’s Hospital Medical Center, Cincinnati, OH, United States; ^13^Division of Behavioral Medicine and Clinical Psychology, Cincinnati Children’s Hospital Medical Center, Cincinnati, OH, United States

**Keywords:** Fragile X Syndrome, neurodynamics, thalamocortical dysrhythmia, auditory entrainment, statistical learning, brain-computer interface

## Abstract

Fragile X Syndrome (FXS), caused by the loss of function of the *Fmr1* gene, is characterized by varying degrees of intellectual disability, autistic features, and sensory hypersensitivity. Despite phenotypic rescue in animal deletion models, clinical trials in humans have been unsuccessful, likely due to the heterogeneous nature of FXS. To uncover the basis of individual- and subgroup-level variation driving treatment failures, we propose to test and modulate thalamocortical drive as a novel “bottom-up” neural probe to understand the mechanics of FXS-relevant circuits. Our study employs trial-level EEG analyses (neurodynamics) to detect fine-grained differences in brain activity using sensory and statistical learning paradigms in children with FXS, autism spectrum disorder (ASD), and typically developing controls. Parallel analysis in the FXS knockout mouse model will clarify its relevance to human FXS subgroups. In a randomized crossover study, we will evaluate the efficacy of closed-loop auditory entrainment, indexed on individual neurodynamic measures, aiming to normalize neural responses and enhance statistical learning performance. We anticipate this approach will yield opportunities to identify more effective early interventions that alter the trajectory of intellectual development in FXS.

## Introduction

Fragile X Syndrome (FXS) is an X-linked monogenetic disorder characterized by reduced Fragile X Messenger Ribonucleoprotein (FMRP) that presents with diverse cognitive and behavioral impairments (Hagerman et al., 2017). Despite the discovery of the silenced Fmr1 gene over three decades ago (Pieretti et al., 1991) and the subsequent development of the *Fmr1*^–/–^ knockout (KO) mouse, translational research has not yet effectively alleviated the core symptoms of the disorder (Berry-Kravis et al., 2018). Notably, therapies that have successfully reversed deficits in *Fmr1*^–/–^ KO models have failed to produce positive results in over a dozen human clinical trials (Erickson et al., 2017; Erickson et al., 2018). We postulate that this translational failure stems from an oversimplified assumption of homogeneity in human FXS research. In stark contrast to the *Fmr1*^–/–^ KO mouse model in which no protein is produced, patients exhibit significant variability in electroencephalographic (EEG), molecular, and behavioral phenotypes, likely reflecting human-specific factors including complex cortical organization and higher-order cognition (Van der Molen et al., 2014; Goswami et al., 2019; Hall et al., 2008; Heard et al., 2014; Jonak et al., 2020). This heterogeneity has contributed to the absence of validated biological markers of the disorder, exposing a key obstacle to therapeutic progress in FXS (Sahin et al., 2018).

Thalamocortical circuits and neural oscillations provide a promising mechanistic framework for addressing this heterogeneity. Thalamocortical networks regulate the rhythmic brain activity that underlies sensory processing and cognitive function, and disruptions in these circuits have been implicated across multiple neuropsychiatric conditions (Choi et al., 2015; Llinas et al., 1999, 2005). Therefore, thalamocortical dysrhythmia may reflect individual differences in the balance between neural mechanisms driving sensory systems and their impact on cognition. Importantly, this dysrhythmia can be measured non-invasively using EEG through alpha oscillations (∼10 Hz), which serve as a proxy for thalamocortical activity (Halgren et al., 2019), and may represent tractable targets for intervention. Brain-computer interfaces (BCI) offer a promising approach for targeting these oscillatory disturbances by continuously monitoring brain activity through EEG, detecting specific neural features like peak alpha frequency (PAF) in real-time, and automatically adjusting stimulation parameters to obtain and maintain desired brain states. This closed-loop approach enables individualized, real-time modulation of thalamocortical activity, potentially addressing the heterogeneity observed in FXS.

**FIGURE 1:**
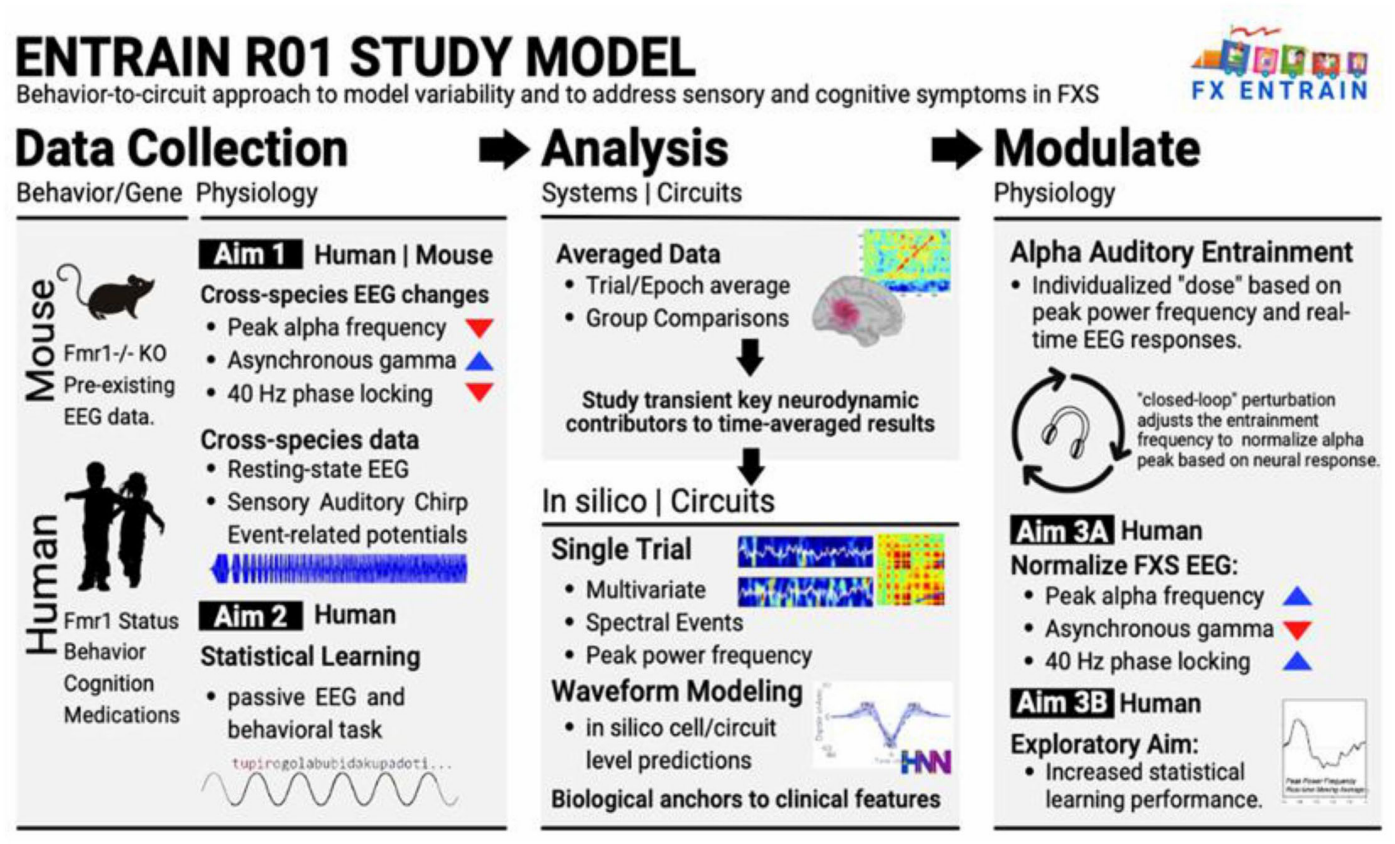
Overview of proposal scientific premise and approach.

This Frontiers in Neuroscience Perspective article uses the FX ENTRAIN study as an illustrative example for understanding and modulating the underlying mechanisms contributing to heterogeneity in FXS by systematically exploring (Figure 1):

(1)    Which specific neurophysiological biomarkers reliably characterize FXS pathophysiology?
(2)    Can novel BCI interventions effectively modulate these biomarkers?
(3)    Does successful biomarker modulation produce measurable functional improvements?

Addressing these questions, our approach targets neurophysiological biomarkers linked to thalamocortical regulation of neural oscillations, a potential source of phenotypic heterogeneity. We will explore these neurodynamics using auditory and pattern-based learning paradigms, modulating these sensory and cognitive systems with individualized auditory entrainment via BCI, resulting in “bottom up” modulation of thalamocortical circuits. In our initial prototype, we will monitor PAF and adjust entrainment frequencies stepwise to obtain a target close to 10 Hz, examining the potential use for these personalized therapeutic interventions for patients with FXS.

## Identifying neural biomarkers in FXS

### EEG as optimal method for non-invasive biomarker detection

Electroencephalographic is a highly feasible method for investigating neurophysiological variability in FXS. Dense-array EEG techniques can provide a real-time examination of brain activity and can be used to localize superficial cortical sources within an error margin less than 1 cm (Song et al., 2015). With the addition of inferior surface data, source localization improves the estimate accuracy for deeper brain regions (Song et al., 2015). In the FX ENTRAIN study, EEG collection will occur at each study visit and will include three primary measures: (1) resting-state EEG, (2) sensory auditory chirp, and (3) passive, pattern-based statistical learning (SL). Across these paradigms, we will examine key biomarkers including alpha and gamma power, PAF, asynchronous gamma activity, 40 Hz phase synchronization [intertrial coherence (ITC)], evoked power, transient spectral events, neural entrainment to structured auditory stimuli, and the word-learning index (WLI). Furthermore, this study will implement trial-level (single-trial) EEG analyses to assess oscillatory activity and phase synchronization on a per-trial basis rather than relying solely on group-averaged effects. This approach allows us to assess variability in brain activity on a trial-by-trial basis, providing a more detailed understanding of neurophysiological heterogeneity in FXS. In addition to quantitative analysis designed around our scientific goals, the study neurologist will review all research EEG tracings for any evidence of epileptic activity (Heard et al., 2014).

### Translational challenges in FXS neurophysiology

Group-level EEG abnormalities are well-established in both humans with FXS and *Fmr1*^–/–^ KO mice, presenting a promising translational bridge. Both populations show increased resting-state gamma power and a reduction in sound-evoked gamma-band synchrony, pointing to similar network hyperexcitability and impaired sensory processing (Wang et al., 2017; Lovelace et al., 2018; Jonak et al., 2022). Furthermore, both human and mouse studies have identified altered event-related potentials (ERPs), such as increased N1 auditory evoked potential amplitudes and reduced habituation to repeated stimuli, which are consistent with auditory hypersensitivity and deficits in sensory gating (Van der Molen et al., 2012; Jonak et al., 2020). While high frequency activity has shown consistent translational connections, exploration of lower frequencies such as alpha is an emerging area of study. During resting-state EEG, individuals with FXS demonstrated a reduction and global leftward shift in alpha, while studies in *Fmr1*^–/–^ KO mice show conflicting results, with some showing no differences from controls and others demonstrating a decrease in alpha power (Pedapati et al., 2022; Jonak et al., 2024; Kozono et al., 2020). Studies using specific auditory stimulation paradigms reveal a more nuanced picture. During an auditory gap-in-noise task, *Fmr1*^–/–^ KO mice showed a significant reduction in theta/alpha band phase synchronization in the primary auditory cortex, demonstrating impediment to temporal processing (Deane et al., 2025). However, during an auditory chirp paradigm, individuals with FXS demonstrated increased alpha synchronization at onset and offset of stimulation, but no change during stimulation (Ethridge et al., 2017). Ultimately, these translational challenges suggest that while group-level effects show key similarities, there remain neurophysiological features in human FXS that are not yet adequately captured by the highly uniform *Fmr1*^–/–^ KO mouse model.

### Thalamocortical dysrhythmia as source of heterogeneity

Thalamocortical dysrhythmia (TCD) provides a unifying framework for these scattered findings. We hypothesize that alterations in thalamocortical activity may be a system-level hypothesis that underlies these EEG findings and is thus far underexplored in FXS and preclinical models. Specifically, TCD is an electrophysiological motif derived from magnetoencephalography and EEG that has been attributed to the dysregulation of cortical excitability and observed across neuropsychiatric conditions (i.e., epilepsy, Parkinson’s disease, tinnitus, depression, and neuropathic pain) (Choi et al., 2015; Llinas et al., 1999, 2005). Similarly to our previous findings in FXS (Pedapati et al., 2022), subgroups that display TCD have reduced alpha power, increased theta power, increased gamma power, and predominance of theta-gamma cross-frequency coupling (CFC), a metric describing frequency band interactions, notably lower frequency regulation of higher frequency activity. Alpha band frequency has been demonstrated to mediate feedback throughout the thalamocortical system (Halgren et al., 2019). Abnormalities in alpha and gamma power demonstrate significant clinical associations with several core features of FXS, including cognitive function, anxiety, social communication, and auditory attention. Our central hypothesis is that disturbances in thalamocortical function, as measured with EEG, alter global alpha (∼10 Hz) and gamma (>30 Hz) activity which in turn impair sensory and cognitive function.

### Transient events as subgroup biomarkers in FXS

Standard EEG analyses mask individual differences by averaging across trials and participants. To address this, the study team has incorporated signal analysis techniques that examine transient, non-continuous features (Jones, 2016) of EEG data to capture inherent neurophysiological variability across males with full mutation (mosaic and non-mosaic) and females with FXS (Baker et al., 2019; Meng et al., 2021; Shaffer et al., 2020; Smith et al., 2021), using trial-by-trial variability measures to assess individual and subgroup differences. A critical lesson from recent success in RCTs in FXS is the utility of targeting subgroups (Berry-Kravis et al., 2018, 2021). Neurodynamic analyses includes non-continuous, trial-by-trial dynamics of oscillatory activity in unaveraged data, and can be applied resting-state and ERP studies (Jones, 2016) and can better reflect individual-level variation of EEG data (Shin et al., 2017). Several distinct time-domain neurodynamics, for example, can lead to a net increase in mean spectral power. Recent studies have shown that neurodynamic features, which may include brief, high-intensity bursts in various frequency bands, have been used for granular behavioral predictions, such as neurocognitive responses (Becker et al., 2020) or predicting sensory thresholds (Shin et al., 2017).

## Task-based paradigms probe functional networks

### Sensory processing through auditory chirp

To process complex sensory patterns, the brain must detect cues and mount precise neural responses. We will identify transient, non-continuous oscillatory features of brain EEG measured responses to auditory evoked potentials (AEPs) to a chirp stimulus in unaveraged trial by trial data at the sensor level. We will test that features of these transient oscillations are associated with individual and subgroup-level variation (including mosaic status and clinical phenotype). In addition, we will compare neurodynamic features of AEPs between *Fmr1*^–/–^ KO and wildtype mice to identify which human subgroup most closely matches the neurodynamic profile of the mouse model. The auditory chirp stimulus probes thalamocortical processing by measuring how neural oscillations synchronize with dynamic frequency changes. This synchrony metric used is intertrial coherence (ITC), also referred to as the phase-locking factor. ITC measures the consistency of the EEG signal’s phase alignment across multiple presentations (trials) of a specific stimulus or pattern element. High ITC indicates that neural oscillations are reliably phase-locked to the input, reflecting a precise, robust neural encoding – essentially, a clear “signal” relative to background activity. Conversely, low ITC suggests variability or temporal jitter in the neural response, indicating a less precise or “noisier” neural representation. Analyzing ITC allows us to move beyond averaged amplitudes and probe the fidelity of neural information processing, foreshadowing the signal-to-noise (S/N) framework discussed later. Individuals with FXS show reduced gamma ITC to the chirp stimuli, indicating impaired fidelity in basic sensory processing (Ethridge et al., 2017, 2019).

### Cognitive processing through statistical learning

Statistical learning (SL) is a fundamental ability to extract patterns and regularities from sensory input through passive exposure, without explicit instruction or conscious effort to learn (Bulf et al., 2011; Krogh et al., 2013). This process is crucial for typical development, particularly in language acquisition for tasks like segmenting words from continuous speech (Saffran, 2018). Despite its importance, SL remains largely unexplored in FXS and has received limited attention in ASD (Obeid et al., 2016; Scott-Van Zeeland et al., 2010). Given that improving communication is a primary goal for many families affected by FXS (Bailey et al., 2008), understanding whether SL is impaired and potentially amenable to intervention holds significant clinical relevance. A major advantage of studying SL is its feasibility; it can be assessed passively using EEG across diverse functional levels and ages, including infants (Choi et al., 2020), overcoming challenges posed by more demanding neurocognitive tests (Budimirovic et al., 2017; Schmitt et al., 2019, 2020; Shaffer et al., 2020).

**FIGURE 2:**
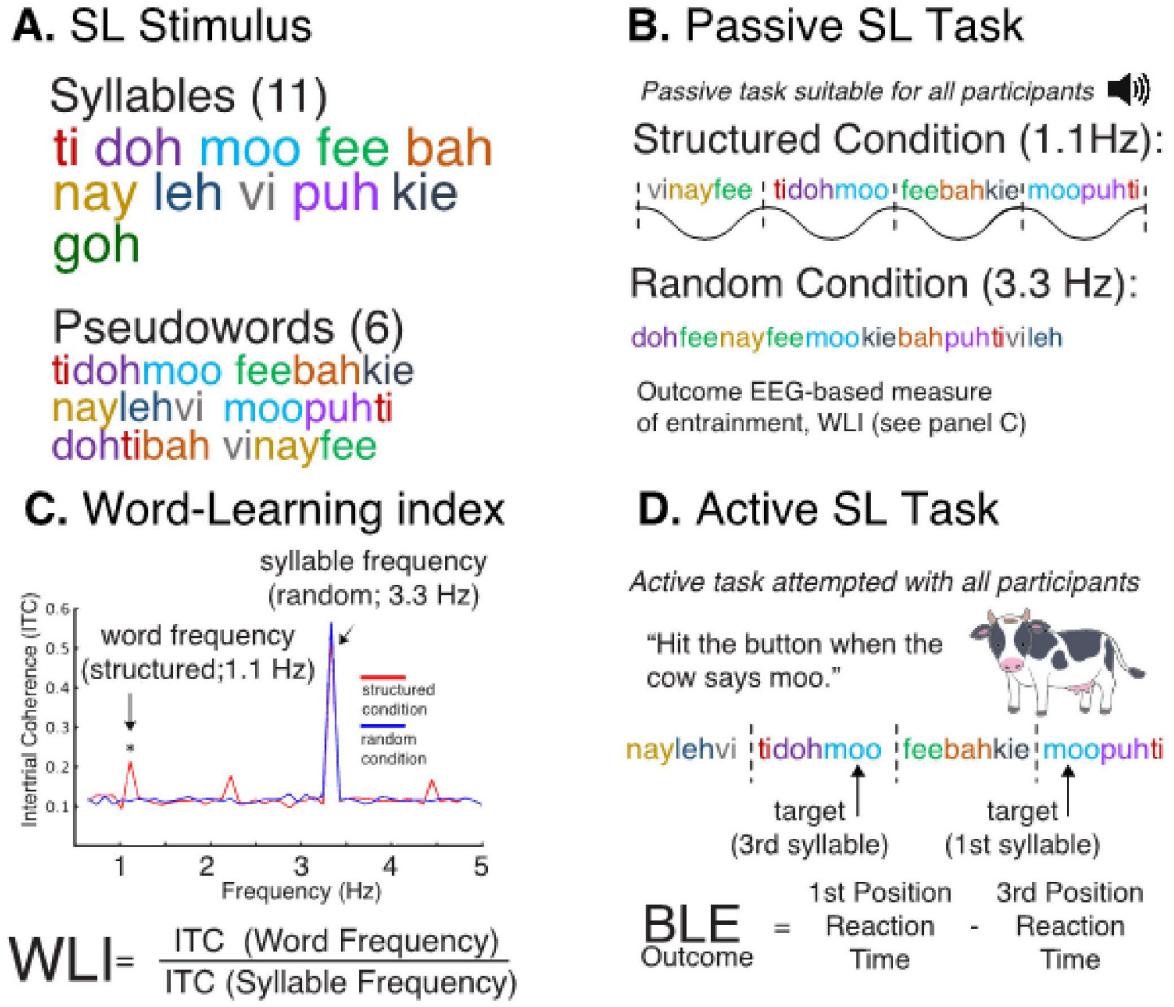
Assessment of statistical learning (SL). **(A)** Eleven synthesized generated syllables are combined into six trisyllabic pseudowords. **(B)** A structured (experimental) and random (control) stream are played to the participant during EEG. **(C)** Word learning index (WLI) is a previously vali-dated EEG-based entrainment measure of learning. Similar our sensory index, it is also based on intertrial coherence (ITC). SL leads to perceptual grouping of trisyllabic words, indexed by a relative increase in phase synchrony at the word presentation rate and a decrease in synchrony at the syllable rate over exposure. **(D)** Behavioral Learning Effect (BLE) is a reaction-time based measure of SL. Participants respond to a target syllable embedded in a continuous auditory stream of pseudowords.

To quantify how the brain tracks statistical patterns and responds to sensory inputs using EEG, our approach focuses on neurodynamic features that reflect the consistency and precision of neural responses on a trial-by-trial basis. Importantly, the neural entrainment that occurs during statistical learning, as the brain implicitly identifies recurring patterns within an auditory stream, like auditory chirp, can also be effectively quantified using ITC (Batterink and Paller, 2017; Batterink, 2017). Derived from ITC, WLI assesses the shift in the ratio of word to syllable frequency, demonstrating an EEG-derived measure of passive pattern-based learning (Figure 2). Therefore, ITC provides a common neurodynamic measure to assess response fidelity in both a controlled sensory paradigm (chirp) and a passive learning task tapping into cognitive processes (SL). This positions SL, quantified via metrics like ITC and WLI, as an ideal and feasible bridge between investigating basic sensory processing deficits and understanding their potential impact on cognitive functions like implicit learning in FXS.

To assess individual differences in neurodynamics, these EEG markers of SL performance will be examined, including neural entrainment, phase synchronization, WLI, and trial-by-trial variability in EEG responses. This approach will provide insight into how the BCI entrainment may help shift alpha oscillations, an indicator of thalamocortical activity, into a more functionally adaptive range, ultimately improving SL performance in individuals with FXS.

## BCI as intervention in sensory and cognitive perturbation studies

### Binaural beats as alpha auditory entrainment stimulation

Our study aims to use data from these sensory and cognitive objectives to parameterize a novel BCI that uses auditory stimuli to enhance alpha oscillations. This approach is designed to test a key question: whether the normalization of targeted EEG activity can lead to improved sensory and SL markers within the disorder. We will employ a non-invasive BCI using auditory binaural beats, referred to as alpha auditory entrainment (AAE) to modulate neural activity. Binaural beats are an established phenomenon in which two tones of slightly different frequencies are presented to each ear, and the brain perceives a beat frequency equal to the difference between the two tones (Ingendoh et al., 2023). This non-invasive, low-risk approach offers a promising way to entrain neural oscillations.

### Modulation of TCD biomarkers

Our BCI intervention will be designed to enhance alpha oscillations, which we have identified as a key component of TCD as a source of heterogeneity in FXS. Specifically, we will use the individual’s PAF to parameterize the BCI, providing a personalized starting point for entrainment. While PAF is our initial target, this framework is designed to be adaptable, allowing for the modulation of other biomarkers, like spectral events. This approach directly ties back to the concept of TCD, as we hypothesize that modulating alpha oscillations will restore the top-down inhibitory control needed to normalize aberrant gamma activity.

### Individualized stimulation to address heterogeneity

A central component of our approach is the use of a closed-loop system. Unlike traditional open-loop interventions, our BCI will be individualized for each participant. By targeting each individual’s unique PAF, we account for the significant heterogeneity that exists in FXS and address the issue of clinical instability observed in previous trials. Further, the closed-loop system allows us to monitor the effect of the auditory binaural beats stimulation in real-time. We can check whether the biomarker is being altered or improved and adjust the stimulation parameters dynamically based on this feedback. This creates a cyclical, informed, and personalized approach to neuromodulation, which will allow us to directly quantify the effect of the BCI intervention by monitoring real-time EEG to see if the alpha rhythms are “paced” into a typical range. Our large collection of preliminary data demonstrates that individuals with FXS have “noisy” asynchronous gamma activity and a marked reduction in alpha power, suggesting altered thalamocortical function. We will test if individualized auditory perturbation compared to a sham condition will result in normalization of these neurodynamic responses. Furthermore, we will test whether this change in EEG activity is directly associated with improvements in statistical learning (SL) behavioral performance for a subset of participants. The results will directly address whether the EEG alterations observed in FXS represent a physiological mechanism that can be tractably targeted, or if they simply reflect compensatory changes.

## Study goals

The scientific goals of FX ENTRAIN are to identify pathophysiological mechanisms, specifically, disruptions in thalamocortical regulation and abnormal neural oscillations, that can be targeted to alleviate core sensory and cognitive impairments in FXS via the following steps: (1) study non-continuous features (source-localized EEG measured neurodynamics) of sensory-driven brain activity to characterize patient-level heterogeneity and constitute group effects; (2) identify neurodynamic features in the *Fmr1*^–/–^ KO which are conserved in patient subgroups; (3) examine neurodynamics associated with statistical learning, which reflects cognitive processes rather than sensory driven responses; (4) develop an individualized controlled closed-loop auditory intervention to modulate brain activity and normalize sensory and cognitive neurodynamics. These efforts have potential to be highly impactful by providing a mechanism for enhancement of early brain-based interventions, which could in turn alter the trajectory of intellectual development in which no definitive treatments are available.

## Study design and methodology

The human studies in this project will consist of a case-control study (Aim 1 and 2) and an acute randomized clinical trial (RCT) (Aim 3). The case-control study will be completed over two visits of approximately 4-h each and separated by approximately 1–4 weeks, including: blood draw for genetic and molecular analyses (if required for genetic testing), medical and psychological assessments, neurocognitive testing, and EEG/AEP procedures. Subjects will be then eligible for a two-visit, randomized controlled, crossover acute perturbation study to investigate the effect of auditory intervention or sham stimulation on neural responses associated with (1) the auditory chirp response and (2) statistical learning. We estimate that the two visits will be approximately 4 h each, separated by a 1–4-week washout. We will account for differences in the two visits by considering sequence in our analysis. In sufficiently cooperative participants, behavioral responses to the SL learning task will be obtained in addition to EEG measures.

The primary neurophysiological measures explored in this study include:

- Resting-state EEG: Alpha and gamma power, PAF, and asynchronous gamma activity.
- Sensory auditory chirp responses: Phase synchronization (40 Hz intertrial coherence), evoked power, and transient spectral events.
- Statistical learning task responses: Neural entrainment to structured auditory stimuli, phase synchronization, and WLI.
- Effects of AAE: Modulation of alpha oscillations, synchronization changes, and impact on sensory and cognitive EEG markers.

For the murine component, we have also setup a collaboration to obtain resting state and auditory evoked potentials to compare neurodynamic features between the *Fmr1*^–/–^ KO model and human subgroups (Lovelace et al., 2018; Jonak et al., 2020). This paper will focus on exclusively describing the human study design.

## Study population

The target enrollment for the case control study is 120 subjects between the ages of 5 and 10 years old. This includes 40 FXS (>200 CGG repeats) and 40 IQ-, sex-, and chronologically aged matched non-syndromic (i.e., idiopathic) ASD controls, and 40 sex- and chronologically aged matched typically developing controls (TDC). Males and females will be recruited in 1:1 ratio for all studies, as FXS females have been understudied despite significant disability. An age and sex matched idiopathic ASD group was included to weaken the inference that genetic liability alone would account for group difference. Moreover, some of our scientific propositions share a common element–diminished PAF (Dickinson et al., 2018)–in both ASD and a monogenic autism-related condition like FXS, which could yield broader insights beyond the specific syndrome.

## Study rigor, data and code availability

The team has worked closely with the study biostatistician to design a valid and robust experimental design to study the major goals. Study data is kept in a secure REDCap database and reviewed by multiple team members to ensure the integrity and minimize biases. EEG and behavioral data are coded and blinded concerning subject and group status during preprocessing for analysis. Similarly, sham or entrainment conditions will be randomized with a blinded key until completion of the study and database lock. The study team will also upload complete, cleaned, de-identified data to the National Database for Autism Research (NDAR) within 9 months of the final year of project funding. All code used for study analysis, including operations and raw files, will be uploaded to the project’s central code repository at http://github.com/cincibrainlab. This will provide transparent access to all study team members and potential peer-reviewers an audit trail of code modifications and the ability to independently reproduce the results.

## Study governance

The FX ENTRAIN investigative team is comprised of a rare combination of preclinical and clinical experts from multiple institutions with a track record of positive working relationships focused on a disease using parallel interrelated approaches to tackle major challenges. FX ENTRAIN was designed to work in concert with a large existing National Institute of Child Health and Human Development (NICHD) Fragile X Center at the home institution. In addition, we have convened a family advisory committee steered by the Director of Research Facilitation and Associate Director at the National Fragile X Foundation to ensure results are effectively disseminated to appropriate stakeholders.

## Progress and future directions

FX ENTRAIN (clinicaltrials.gov: NCT06227780) was opened on 5/2023 and is actively enrolling subjects with a estimated study completion date of 5/2028. We have collected pilot data on 19 participants which is being used to optimize the BCI intervention for the RCT.

## Conclusion

Addressing heterogeneity in FXS, FX ENTRAIN employs neurodynamics to analyze individual variability in EEG signatures linked to sensory and cognitive disturbances. Non-invasive perturbation is used critically, both to test causal influences of brain activity on function and to probe underlying circuit regulation. This innovative, collaborative research aims to translate understanding of neurodynamic targets into personalized, effective treatments for cognitive symptoms in FXS.

## Data Availability

Publicly available datasets were analyzed in this study. This data can be found here: http://github.com/cincibrainlab.
